# The effect of exogenous calcium on cucumber fruit quality, photosynthesis, chlorophyll fluorescence, and fast chlorophyll fluorescence during the fruiting period under hypoxic stress

**DOI:** 10.1186/s12870-018-1393-3

**Published:** 2018-09-04

**Authors:** Lizhong He, Li Yu, Bin Li, Nanshan Du, Shirong Guo

**Affiliations:** 1 Shanghai Key Lab of Protected Horticulture Technology, Horticultural Research Institute, Shanghai Academy of Agricultural Science, Shanghai, 201106 China; 2 Key Laboratory of Southern Vegetable Crop Genetic Improvement, Ministry of Agricultural, College of Horticulture, Nanjing Agricultural University, Nanjing, 210095 China; 3 College of Horticulture Shanxi Agriculture University, Taigu, 030801 Shanxi China; 4 Department of Horticulture, Henan Agricultural University, Zhengzhou, 450000 Henan China

**Keywords:** Cucumber, Exogenous calcium, Hypoxic stress, Photosynthesis, Chlorophyll fluorescence, Fast chlorophyll fluorescence

## Abstract

**Background:**

Plants often suffer from hypoxic stress during waterlogging and hydroponic culturing. This study investigated the response of cucumber (*Cucumis sativus* L.) plant growth parameters, leaf photosynthesis, chlorophyll fluorescence, fast chlorophyll a fluorescence transient (OJIP), and fruit quality parameters to hypoxic stress alleviated by exogenous calcium. During the fruiting period, cucumber plants were exposed to hypoxia and hypoxia + Ca^2+^ treatment (4 mM Ca^2+^) for 9 d.

**Result:**

Exogenous calcium application enhanced the biomass and fruit quality of hypoxic stressed cucumber and also increased the net photosynthesis rate, stomatal conductance, intercellular CO_2_ concentration, maximum quantum efficiency of photosystem II photochemistry, actual photochemical efficiency of PSII, photochemical quenching coefficient, and non-photochemical quenching coefficient. Additionally, measurement of chlorophyll a fluorescence transients showed the positive K- and L-bands were more pronounced in leaves treated with hypoxia compared with those with hypoxia + Ca^2+^, indicating that hypoxic treatment induced uncoupling of the oxygen-evolving complex and inhibited electron transport beyond plastoquinone pool (Q_a_, Q_b_) including possible constraints on the reduction of end electron acceptors of photosystem I. Exogenous calcium can reduce these stress-induced damages in cucumber.

**Conclusion:**

This research focused the effect of exogenous calcium on cucumber photosynthesis during the fruiting period under hypoxic stress. Hypoxic stress might impair the photosynthetic electron-transport chain from the donor side of PSII up to the reduction of end acceptors of PSI, and exogenous calcium enhanced electron transport capacity and reduced hypoxic damage of cucumber leaves.

## Background

Land plants are often subjected to low oxygen concentrations in specific tissues during their development and in response to decreased environmental oxygen availability caused by waterlogging, flooding, and hydroponic culturing [1]. Approximately 16% of fertile land worldwide is affected by soil waterlogging, resulting in crop yield reductions of up to 80% [2]. Plants can survive in low oxygen (O_2_) environments through the activation of diverse molecular, metabolic, and physiological responses [3], such as decreases in cellular energy change, a drop in cytoplasmic pH, enhanced aerenchyma formation, stem elongation, the use of gas films around submerged leaves, and the accumulation of toxic end-products from anaerobic respiration and reactive oxygen species (ROS) [4, 5]. Plant growth, flash and dry matter accumulation are significantly depressed by root zone oxygen deficiency [6]. Additionally, hypoxic stress can also destroy the ion transport processes, cell metabolism, and nutrient acquisition, through limits the supply of ATP to plant H^+^-ATPase pumps [7, 8].

Calcium, especially cytosolic free Ca^2+^, has been widely recognized as a key signal molecule in plants and is involved in multiple signal transduction pathways, which mediate plant adaptive responses to abiotic and biotic stimuli [9]. The involvement of calcium in hypoxia responses has been observed in many plants such as rice, wheat, and cucumber [10, 11]. This hypoxia-mediated elevation of Ca^2+^ is fundamental for the activation of genes and synthesis of proteins required for acclimation responses at the cellular, tissue, and organismal levels [6, 12]. The complex processes of fruit growth and development are regulated by genes and metabolic pathways [13]. Photosynthetic carbon assimilation is a key plant metabolic process that is strongly influenced by environmental conditions [14]. Green plants need PSII to absorb energy from sunlight to support fruit development and ripening, but harnessing this tremendous light energy during photosynthesis carries great risk [15], especially when plants suffered from environmental stresses such as salinity [16] and heat stress [17].

Calcium is necessary for plant stress tolerance and proper photosynthetic function through maintaining the membrane stability, osmotic homeostasis and cell signaling [18]. Measuring chlorophyll fluorescence is a powerful and non-invasive technique to determine PSII activity. The abiotic and biotic factors have a significant effect on activity of PSII, so the measurement of PSII can give us a better understanding about plant responses to environmental change and the photosynthetic mechanisms [19]. The most common method is based on high-frequency records of PAM fluorometry emitted by dark-adapted leaves during short (usually one second long) pulses of strong actinic light by a fluorimeter [14]. Fluorescence kinetics can reflect some valuable information about photosynthesis, such as the photochemical efficiency and the function and structure of the photosynthetic electron transport, mainly in PSII [20]. The fluorescence value rises from the initial (F_0_) to the maximal (F_m_) in seconds and can be separated into O, J, I and P phase. The JIP-test, as a mathematical model of the polyphasic transient fluorescence [14], enables measurement of some biophysical parameters and probabilities characterizing the functional and structural attributes of components involved in PSII. Previous reviews had reported that the Ca^2+^ signals contribute to red light, blue light, UV-B signaling and circadian clock of plant [21]. It was hypothesized exogenous calcium application would be associated increased photosynthesis, larger fruits, increased chlorophyll parameters.

Cucumber, one of the largest vegetable crops globally in terms of production, is an economically important vegetable crop and is sensitive to hypoxic stress. Previous studies have demonstrated that exogenous putrescine and 24-epibrassinolide improved the photosynthetic performance of cucumber under salt [16] and Ca(NO_3_)_2_ stress [22], respectively. Examples of the application of fast chlorophyll fluorescence kinetics can also be found in citrus [23], maize, and tomato [14, 24]. However, no studies have yet combined the determination of photosynthetic characteristics with the chlorophyll fluorescence of cucumber under hypoxic stress. Therefore, the aim of this work was to clarify the effect of exogenous calcium on the improvement of photosynthetic performance and fast chlorophyll fluorescence records in fruiting cucumber plants under hypoxic stress.

## Methods

### Plant materials and growth conditions

Cucumber (*Cucumis sativus* L. cv. Jinchun No. 2, hypoxia-sensitive [25]) were sown in plastic trays (41 × 41 × 5 cm) containing quartz sand, and cultured in a greenhouse (32°02′ N, 118°46′ E, Nanjing, China) at 25 °C–30 °C (day) and 15 °C–18 °C (night) under natural light (maximum photosynthesis photon flux density (PPFD) about 1200 μmol m^− 2^ s^− 1^) with relative humidity (RH) from 70 to 85%. Treatments consisted of [8]: 1) Control: half-strength Hoagland solution (containing 2 mM Ca^2+^) with a dissolved oxygen (DO) level of 8.0 ± 0.2 mg L^− 1^; 2) Hypoxia treatment: half-strength Hoagland solution (containing 2 mM Ca^2+^) with a DO level of 1.0 ± 0.1 mg L^− 1^ that was prepared by pumping N_2_ gas into the nutrient solutions; 3) Hypoxia + CaCl_2_ treatment: half-strength Hoagland solution + 4 mM CaCl_2_ with a DO level of 1.0 ± 0.1 mg L^− 1^ and the oxygen concentration in the nutrient solution controlled as in the hypoxia treatment. The oxygen concentration in the nutrient solutions was monitored with an automatic DO control system (Quantum-25, Quantum Analytical Instruments Inc., USA). Every treatment had 18 plants with 3 replicates, and the experimental treatments started when second female flowers of cucumber plants finished fruit setting.

Following 9 d of treatment, the shoots, roots, and fruit of the control and treated plants were harvested, immediately frozen in liquid nitrogen, and stored at − 80 °C for further analysis. Photosynthetic characteristics and chlorophyll a fluorescence transient was measured as indicated below before plants were destructively harvested.

### Measurements of biomass and the quality of cucumber fruits

Plant height, stem diameter, and fruit weight were determined using a ruler, Vernier caliper, and electronic scale, respectively. To determine the fresh weight of stems and roots, the plants were washed with distilled water and weighed after wiping off the water. Fruit soluble protein was measured according to Bradford [26]. Fruit Ca^2+^ content was measured using the Calcium Colorimetric Assay Kit (Bio Vision, Mountain View, CA, USA) following the manufacturer’s instructions [8]. The total soluble solids content (TSS, in °Brix), the titratable acidity (TA, milliequivalents of acid per 100 g of fresh matter) and total soluble sugars of cucumber fruits were assayed in according to Kang et al. [27].

### Measurement of gas-exchange parameters

The net photosynthetic rate (*P*_n_), stomatal conductance (*g*_s_), intercellular CO_2_ concentration (*C*_i_), and transpiration rate (*T*_r_) of the third fully expanded leaf from the shoot tip were monitored using a portable photosynthesis system (Li-6400; LI-COR, Inc., Lincoln, NE, USA) at 10:30 am after 9 d of treatments. Cuvette conditions were maintained at a photosynthetic photon flux density (PPFD) of 1000 μmol photons m^− 2^ s^− 1^, relative humidity at 60–70%, leaf temperature of 25 °C, and external CO_2_ concentration of 380 ± 10 μmol mol^− 1^. Water use efficiency (WUE) was calculated as WUE = *P*_n_*/T*_r_.

### Analysis of chlorophyll fluorescence

Chlorophyll fluorescence imaging of cucumber leaves was performed using an imaging-PAM fluorometer (Walz, Effeltrich, Germany). Leaves were placed in darkness for 30 min prior to measurement. Maximum quantum yield of PSII (Fv/Fm), actual photochemical efficiency of PSII (ΦPSII), photochemical quenching coefficient (qP), and non-photochemical quenching coefficient (NPQ (=Fm/Fm′-1)) were measured and calculated in according to Lu et al. [28] and Yuan et al. [16]. The PAM software selected the same areas of each leaf for the fluorescence image.

### Measurement of chlorophyll a fluorescence transients

Chlorophyll a fluorescence (OJIP) transients were measured using a Handy Plant Efficiency Analyzer (Handy-PEA, Hansatech Instruments Ltd., Norfolk, UK) according to the method of Strasserf and Srivastava [29]. All measurements were done with plants that had been dark-adapted for 3 h at room temperature (22–25 °C). Transient fluorescence was induced by approximately 2000 μmol m^− 2^ s^− 1^ red light provided by an array of three light-emitting diodes (peak 650 nm) that focused on the leaf surface to give homogenous illumination over the exposed area of the leaf (4 mm in diameter). Data were sampled at 10 μs intervals for the first 300 μs, providing excellent time resolution of *F*_*0*_ and the initial rise kinetics. The time resolution of digitization was then switched to slower acquisition rates as the kinetics of the fluorescence signal slowed.

OJIP transient was analyzed according to the JIP-test formulae [23, 30]. The fluorescence intensity at 20 μs (considered to be minimum fluorescence F0); maximal fluorescence intensity equal to Fm as the intensity was high enough to ensure the closure of all reaction centers (RCs) of PSII; and fluorescence intensity at 300 μs (F300 μs), 2 ms (J-step, FJ), and 30 ms (I-step, FI) [30]. The following parameters that all refer to time 0 (start of fluorescence induction) are: (a) the specific energy fluxes (per reaction center, RC) for absorption (ABS/RC), trapping (TR_o_/RC), electron transport (ET_o_/RC), and dissipation at the level of the antenna chlorophylls (DI_o_/RC) and (b) normalized total complementary area above the OJIP transient or total electron carriers per RC (Sm = EC_0_/RC = Area/(F_m_-F_0_)). Approximated initial slope of the fluorescence transient *f*(t): M_0_ = 4 M_o_ = 4 • (F_300μs_-F_0_)/(F_m_-F_o_). Performance index (PI) on absorption basis PI_(abs)_ = (RC/ABS) • [φP_o_/(1– φP_o_)] [ψ_o_ /(1– ψ_o_)]. Maximum quantum yield of primary photochemistry: φP_o_ = F_v_/F_m_ = (F_m_ - F_o_)/F_m_.

Extended analysis of OJIP transients was done by calculating the relative variable fluorescence: V_t_ = (F_t_ - F_o_)/(F_m_ - F_o_), W_K_ = (F_t_ - F_o_)/(F_300 μs_ - F_o_) and the differences between the treated and control samples. The ΔL-, ΔK- and ΔJ-bands appear around 130, 300, and 2 ms, respectively, and are associated with the ungrouping of PSII units, the uncoupling of the oxygen-evolving complex (OEC), and the accumulation of Q_A_^−^ [23].

### Statistical analysis

Experimental data were processed with SAS software (SAS Institute, Cary, NC, USA) using Duncan’s multiple range test at the *p* < 0.05 level of significance.

## Results

### Morphological parameters and the quality of fruit

Plant height, stem diameter, and fresh weight of shoot and root decreased significantly under hypoxic treatment (*p* = 0.05), especially root fresh weight (Table 1). Plant weight, stem diameter, and shoot and root fresh weight were 18% to 49% of the control. Conversely, exogenous calcium alleviated hypoxic stress-induced inhibition of growth, but exogenous calcium exerted no effect on stem diameter.

**Table 1:** Effect of exogenous calcium on biomass of cucumber at fruiting period under hypoxia stress

Treatment	Plant height (cm)	Stem diameter (mm)	Fresh weight of shoot (g plant^−1^)	Fresh weight of root (g plant^−1^)
Control	127.43 ± 1.78 a	14.11 ± 1.12 a	244.95 ± 19.06 a	88.73 ± 1.46 a
Hypoxia	104.33 ± 2.07 c	11.29 ± 0.29 b	171.06 ± 25.65 b	45.33 ± 5.92 c
Hypoxia + Ca^2+^	115.96 ± 3.92 b	12.72 ± 0.51 ab	216.31 ± 12.35 a	60.44 ± 8.85 b

Each value is the mean ± SE of three independent experiments. Different letters indicate significant differences within columns at *p* = 0.05, according to Duncan’s multiple range test

**Fig. 1:**
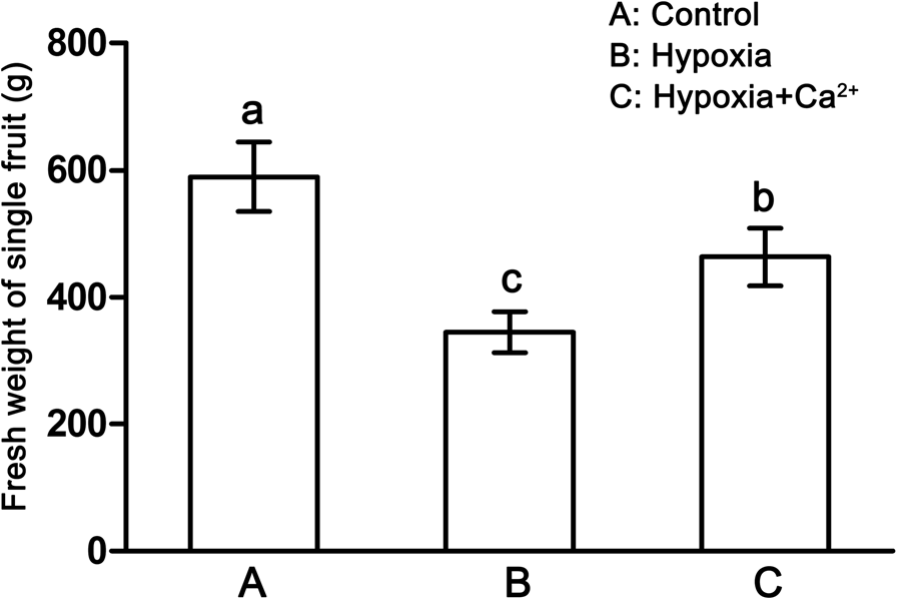
The fresh weight of single fruit of cucumber plants under normoxic conditions (**a**), hypoxic treatments (**b**) and hypoxia + Ca^2+^ treatments (**c**). Values are means ± SE of three independent experiments. Bars marked with different letters are significantly different from each other according to Duncan’s multiple range test (*p* = 0.05)

Fresh weight of fruit was inhibited by hypoxic treatment compared to control treatment (*p* = 0.005), and was higher in the hypoxia + CaCl_2_ treatment compared to the hypoxic treatment (Fig. 1). Although the fresh weight, total protein content, and Ca^2+^ content of fruit decreased under hypoxic treatment, total soluble solids content and soluble sugar concentrations tended to increase (Table 2). Titratable acidity (TA) showed no significant change compared with the control treatment. After treatment with exogenous calcium, fresh weight, Ca^2+^ content, titratable acidity, and soluble sugar concentrations increased markedly when compared with the hypoxic treatment (Table 2).

**Table 2:** The effect of exogenous calcium on fruit quality of cucumber under hypoxia stress

Treatment	Protein content (mg g^− 1^ FW)	Soluble solids	Titratable acidity (%)	Total soluble sugar (%)
Control	589.78 ± 54.45 a	2.53 ± 0.49 b	0.067 ± 0.006 b	0.535 ± 0.084 c
Hypoxia	345.05 ± 32.57 b	3.55 ± 0.61 a	0.052 ± 0.011 b	0.814 ± 0.142 b
Hypoxia+Ca^2+^	463.58 ± 45.53 ab	2.63 ± 0.15 ab	0.093 ± 0.015 a	1.886 ± 0.068 a

Each value is the mean ± SE of three independent experiments. Different letters indicate significant difference at *p* = 0.05, according to Duncan’s multiple range test

### Gas exchange parameters

**Fig. 2:**
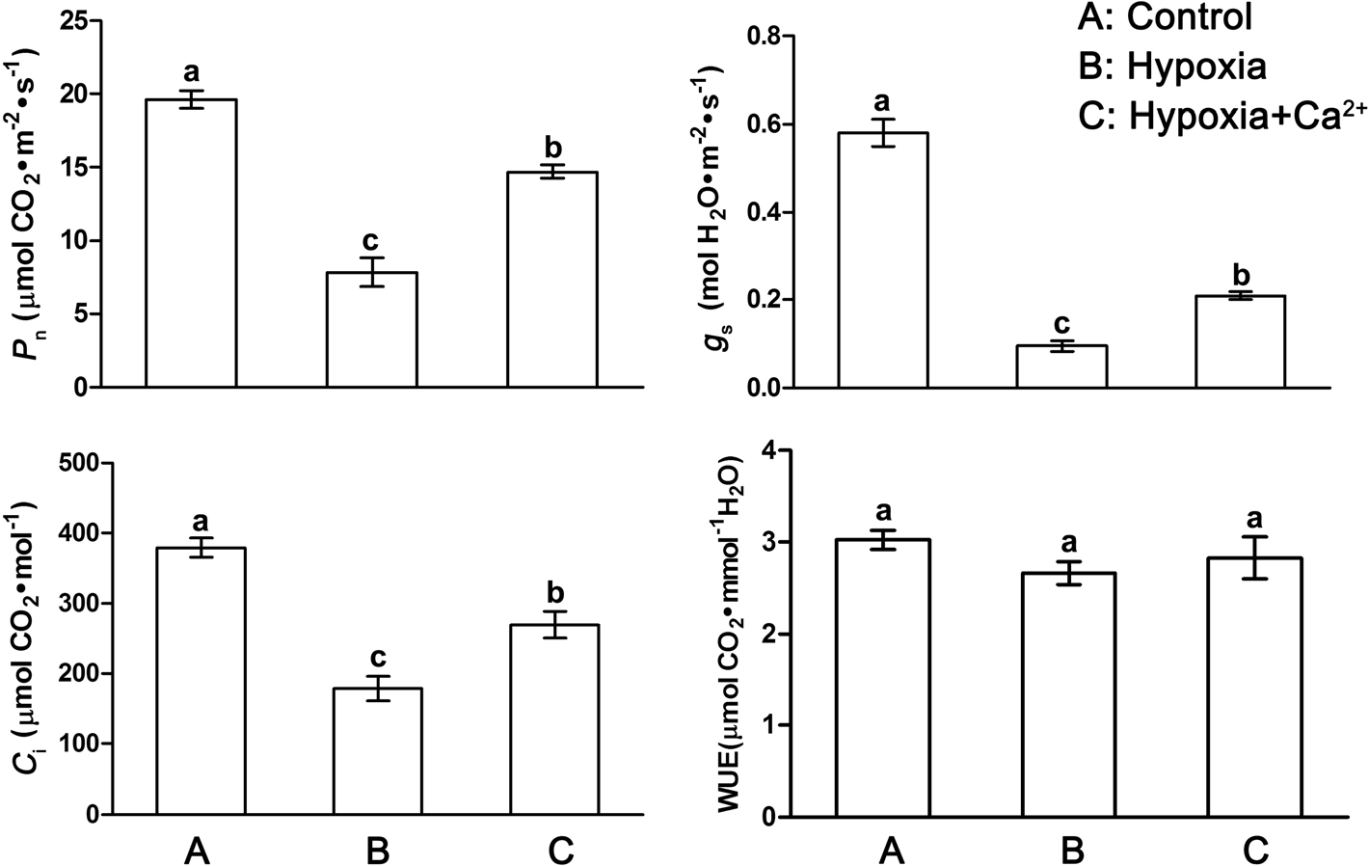
Effect of exogenous Ca^2+^ on gas exchange parameters in leaves of cucumber under normoxic conditions (**a**), hypoxic treatments (**b**) and hypoxia + Ca^2+^ treatments (**c**). Values are means ± SE of three independent experiments. Bars marked with dissimilar letters are significantly different from each other according to Duncan’s multiple range tests (*p* < 0.05). *P*_n_, net photosynthetic rate; *g*_s_, stomatal conductance; *C*_i_, intercellular CO_2_ concentration; WUE, water use efficiency

Hypoxic stress reduced *P*_n_, *g*_s,_ and *C*i to 18 to 47% of control levels (Fig. 2). However, hypoxia-induced negative effects were significantly diminished by exogenous calcium; this increased *P*_n_, *g*_s,_ and *C*_i_ by 51 to 119%, compared with the hypoxic treatment, but did not result in recovery to control levels. WUE did not differ significantly between treatments.

### Chlorophyll fluorescence

**Fig. 3:**
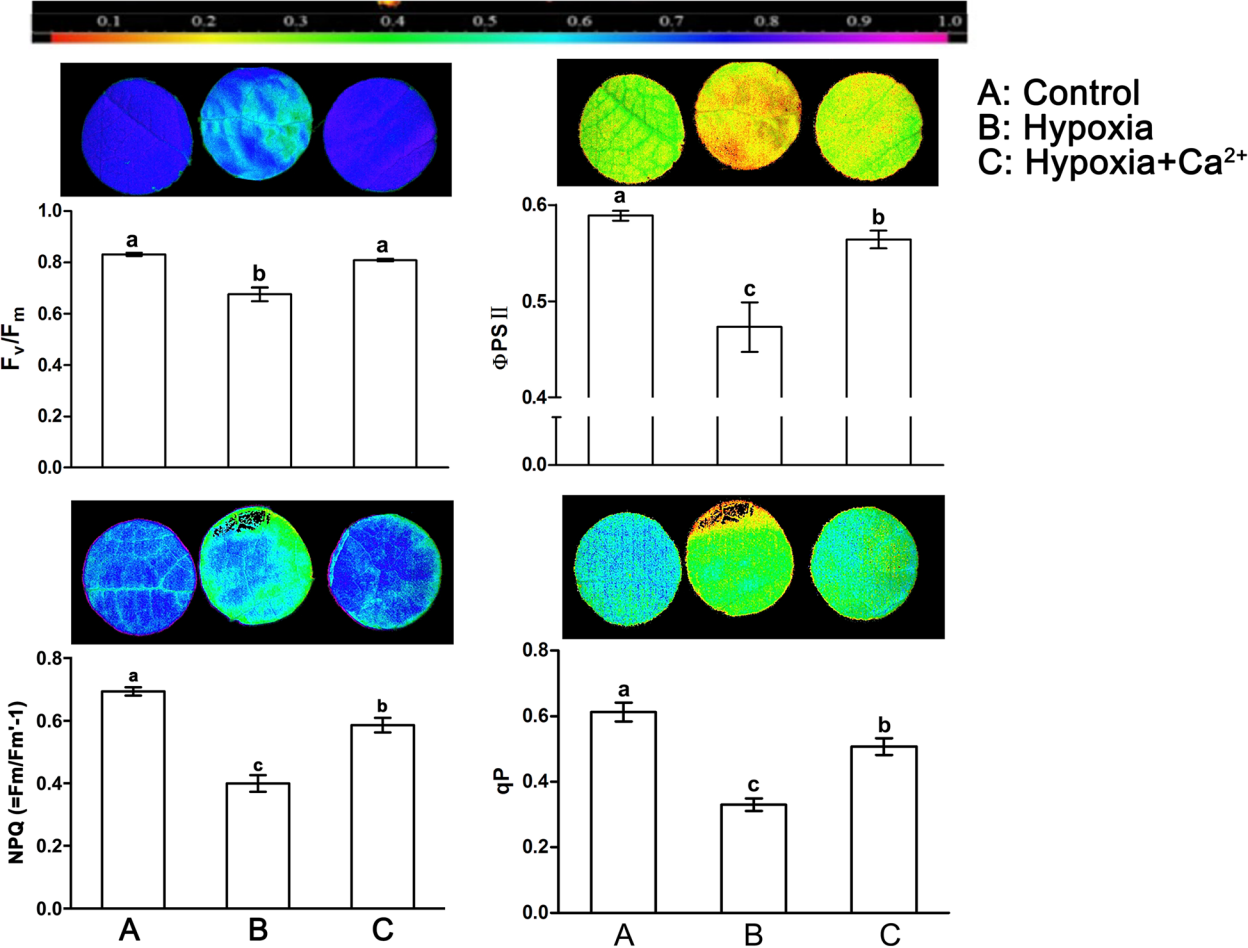
Effect of exogenous Ca^2+^ on chlorophyll fluorescence parameters and images in leaves of cucumber under normoxic conditions (**a**), hypoxic treatments (**b**) and hypoxia + Ca^2+^ treatments (**c**). Values are means ± SE of three independent experiments. Bars marked with dissimilar letters are significantly different from each other according to Duncan’s multiple range tests (*p* < 0.05). Image of Fv/Fm, ΦPSII, qP and NPQ (=Fm/Fm′-1) with actinic illumination of 450 μmol photons m^− 2^ s^− 1^ are shown. Fv/Fm, the maximum quantum yield of PSII; ΦPSII, actual photochemical efficiency of PSII; qP, photochemical quenching coefficient; NPQ (=Fm/Fm′-1), non-photochemical quenching coefficient. Each image in the same column represents the same leaf. The color scale at the top indicates values from 0 (black) to 1 (pink)

Compared with the control, hypoxic treatment significantly decreased Fv/Fm, ΦPSII, qP, and NPQ (=Fm/Fm′-1). Fv/Fm, ΦPSII, qP, and NPQ (=Fm/Fm′-1) were 81.5 to 57.5% of control levels (Fig. 3). Conversely, application of exogenous calcium increased the level of these parameters when compared with the hypoxic treatment (*p* = 0.05). Respective pseudo-color images of leaves indicated the status of the four parameters under different treatments (Fig. 3). The values of Fv/Fm, ΦPSII, qP, and NPQ (=Fm/Fm′-1) across the leaf surface decreased unevenly under hypoxia. Fv/Fm and NPQ (=Fm/Fm′-1) near the veins decreased significantly compared with the control. Exogenous calcium could recover the color of leaves almost to the level of the controls.

### Leaf chlorophyll a fluorescence (OJPI) transients and related parameters

**Fig. 4:**
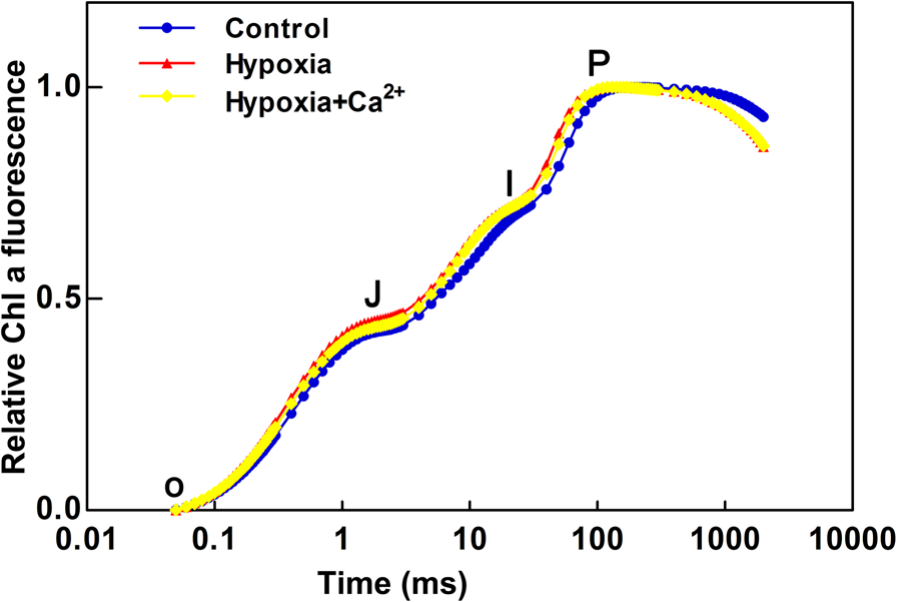
Fast chl a fluorescence transient (OJIP) plotted on logarithmic time scale (0.01–1 s) measured under normoxic conditions (Control), hypoxic treatments (Hypoxia), hypoxia + Ca^2+^ treatments (Hypoxia+Ca^2+^)

All treatments displayed a typical polyphasic increase in OJIP transient, including the O, J, I, and P phases (Fig. 4). Hypoxic stress induced the OJIP transients with a rise at J-step and I-step, but a significant depression at P-step.

**Fig. 5:**
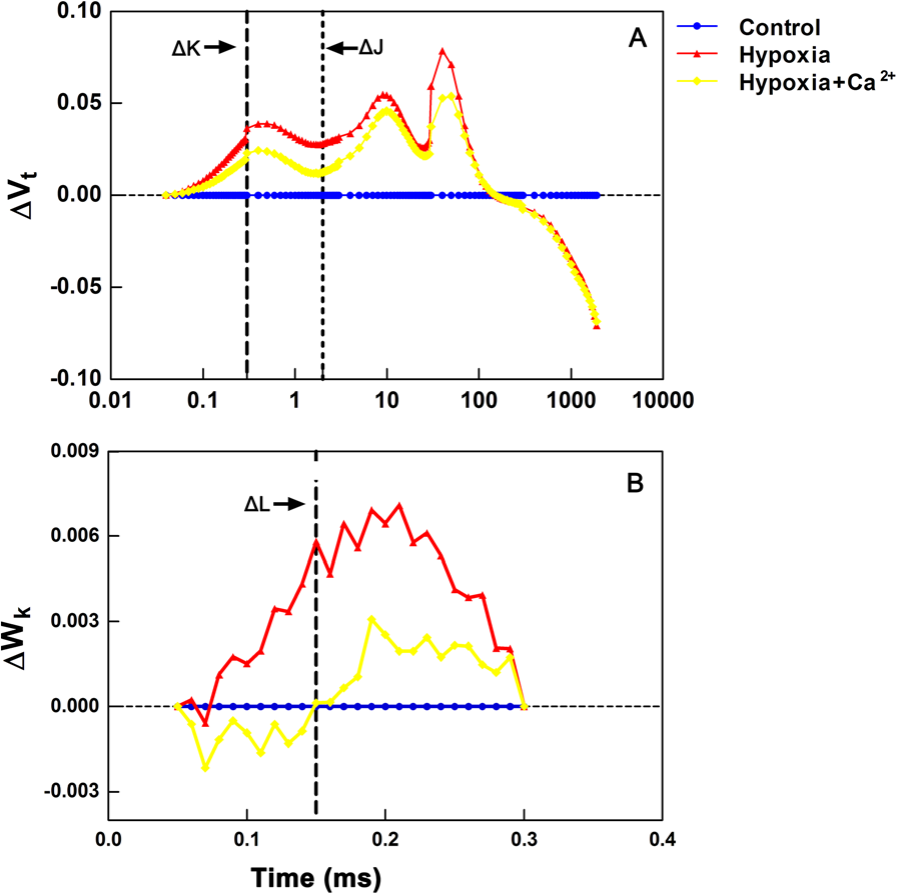
Effect of exogenous Ca^2+^ on relative variable fluorescence ∆V_t_ and ∆W_k_ in leaves of cucumber under hypoxia. Control: normoxic conditions, Hypoxia: hypoxic treatments, Hypoxia + Ca^2+^: hypoxia + Ca^2+^ treatments

The OJIP transient presented in Fig. 5a and b shows differences in variable fluorescence curves ΔV_t_ and ΔW_k_, respectively. There are three distinct trends: i) an increase in the ΔK-band (300 μs), ii) An increase in the ΔJ-band (2 ms), and iii) an increase in the ΔI-band (30 ms) (Fig. 5a). The positive ΔK-band, ΔJ-band, and ΔI-band were more pronounced in hypoxic stressed leaves than in those treated with exogenous calcium. There is a clear ΔL-band that was more pronounced in hypoxic leaves than in exogenous calcium-treated ones (Fig. 5b).

**Fig. 6:**
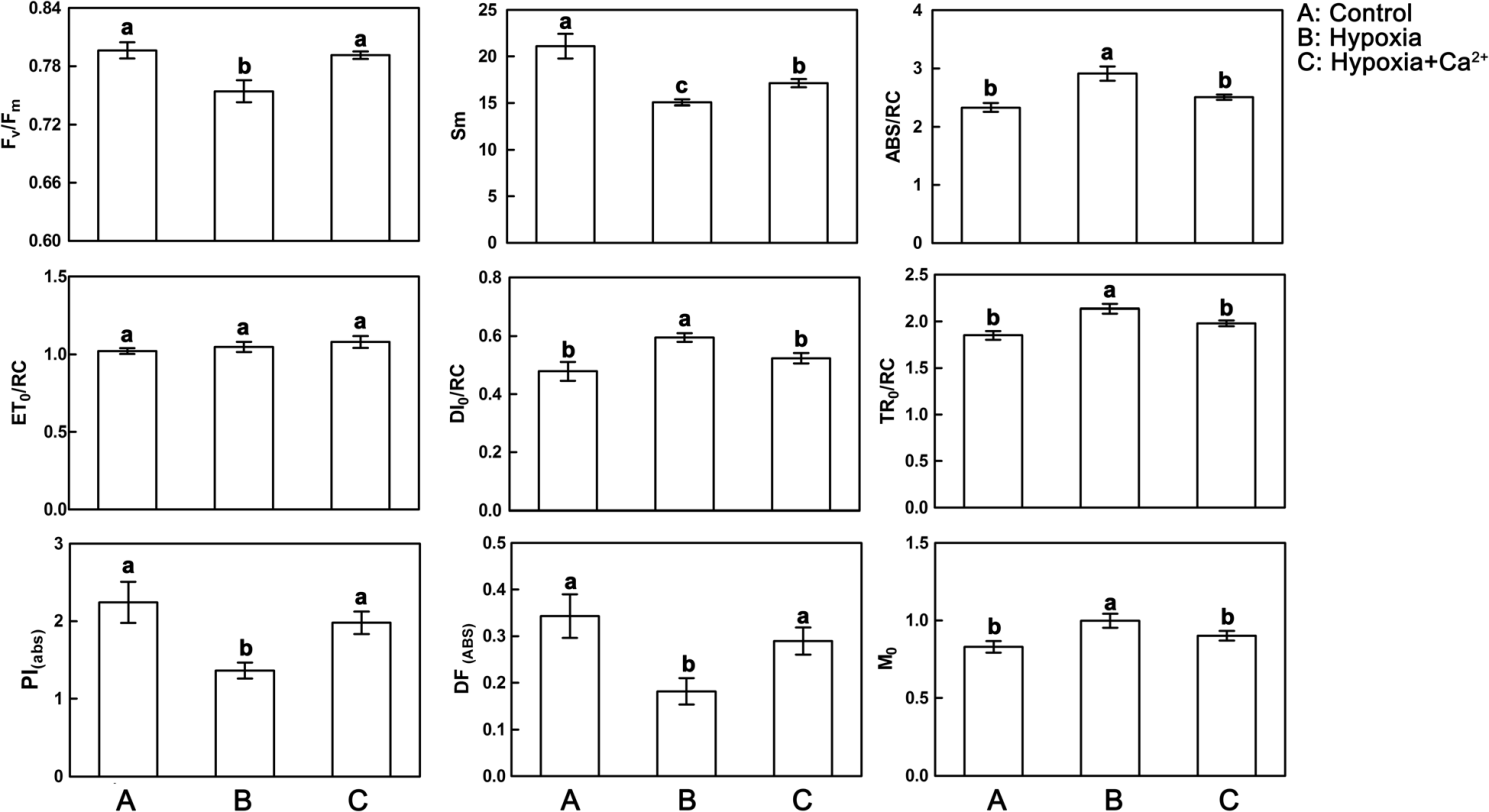
Effect of exogenous Ca^2+^ on JIP-test parameters in leaves of cucumber under normoxic conditions (**a**), hypoxic treatments (**b**) and hypoxia + Ca^2+^ treatments (**c**). Values are means ± SE of three independent experiments. Bars marked with dissimilar letters are significantly different from each other according to Duncan’s multiple range tests (*p* < 0.05). Fv/Fm, the maximum quantum yield of PSII; Sm, total electron carriers per RC; ABS/RC, the specific energy fluxes (per reaction center, RC) for absorption; ET_o_/RC, the specific energy fluxes (per reaction center, RC) for electron transport; DI_o_/RC, dissipation at the level of the antenna chlorophylls; TR_o_/RC: the specific energy fluxes (per reaction center, RC) for trapping. Performance index (PI) on absorption basis PI_(abs)_ = (RC/ABS) • [φP_o_/(1– φP_o_)] [ψ_o_ /(1– ψ_o_)]; Approximated initial slope of the fluorescence transient *f*(t): M_0_ = 4 M_o_ = 4 • (F_300μs_-F_0_)/(F_m_-F_o_)

Compared with controls, hypoxic stressed leaves had significant increases in dissipated energy per RC (DI_0_/RC), trapped energy flux per RC (TR_0_/RC), and absorption flux per RC (ABS/RC) (Fig. 6). Hypoxic stress also decreased total electron carriers per reaction center (S_m_), suggesting that the probability of electron transport beyond Q_A_^−^ was decreased (Fig. 6). This condition finally induced the increased maximum reduction speed of Q_A_^−^ (M_0_) and decreased the dissipated energy flux per RC (DI_0_/RC), maximum yield of primary photochemistry of PSII (Fv/Fm), and performance index on absorption basis (PI_abs_). After applying the exogenous calcium, these performance parameters recovered to control levels (Fig. 6).

## Discussion

Molecular oxygen is involved in a wide variety of plant biochemical reactions. It has also been well documented that plants may reduce their growth, yield, and photosynthetic efficiency as an avoidance strategy in response to a variety of stresses [16, 22, 31]. Thus, studying the response of cucumber plants at fruiting stage to hypoxic stress may help us to understand the mechanisms underlying cucumber hypoxic tolerance. In the present work, hypoxic stress significantly suppressed the growth and photosynthesis of cucumber plants (Table 1, Fig. 2) and reduced cucumber fruit fresh weight (Fig. 1). Exogenous calcium enhanced the growth, photosynthesis and fruit quality of hypoxic stressed cucumbers.

It is widely accepted that phloem transport is inhibited by hypoxia; this is probably caused by decreased sugar importing or reloading into phloem resulting from energy deprivation in the tissues [32]. Kang et al. [27] found that the total soluble and starch content of cucumber leaves increased significantly under hypoxic stress because of decreased sugar transport from leaves to roots. Our previous study also found that root-zone hypoxic stress decreased the biomass of plants and soluble protein of leaves [33]. We obtained similar results in cucumber fruit in the current study. Hypoxia reduced the soluble protein and Ca^2+^ content of cucumber fruit but increased the soluble solids and total soluble sugar (Table 2). After extra Ca^2+^ was added to the nutrient solution, the above indexes approached control levels, indicating that exogenous Ca^2+^ may alleviate hypoxic stress in cucumber plants.

Reduced CO_2_ assimilation is a common response to stress conditions that occurs as a result of stomatal closure and causes further damage to the photosynthetic apparatus. In this work, hypoxia caused a reduction of *P*_n_, *g*_s_, and *C*_i_ in cucumber plants (Fig. 2). The simultaneous decreases in *g*_s_ and *C*_i_ indicate stomatal closure is limiting photosynthesis by limiting access to CO_2_ in the leaf. Conversely, exogenous calcium caused significant increases in *P*_n_, *g*_s_, and *C*_i_ (Fig. 2). Exogenous calcium improved the photosynthetic capacity by enhancing the carbon assimilation capacity of leaves and by regulating stomatal movement under hypoxic stress.

In leaf studies, it is natural to extend the interpretation of chlorophyll fluorescence data to analyze its impact on photosynthetic rates of CO_2_ assimilation [18]. Imaging of chlorophyll fluorescence is becoming increasingly popular as a screening and diagnostic tool [34] and can enhance our understanding of photosynthetic heterogeneity arising from patchy responses of stomata and distributed metabolic regulation [35]. This method can also overcome the disadvantages of conventional chlorophyll fluorescence analysis based on point measurements. Hypoxia, like other abiotic stress, caused injuries across the whole leaf generally, and then decreased the photosynthetic capacity of the injured areas of leaves. To quantify the photosynthetic capacity of stressed cucumber leaves under dark-adapted and light-adapted conditions, we measured Fv/Fm and ΦPSII, respectively [35]. Our results showed that Fv/Fm and ΦPSII were reduced under hypoxic treatment (Fig. 3), suggesting that electron transfer from the primary acceptor plastoquinone (Q_A_) to the secondary acceptor plastoquinone (Q_B_) at the acceptor side of PSII was blocked under stress condition [36]. However, exogenous calcium significantly enhanced Fv/Fm and ΦPSII under stressed condition, indicating that exogenous calcium alleviated the photo-inhibition and improved the photochemical efficiency of stressed cucumber plants [37]. Additionally, exogenous calcium treatments also increased the non-photochemical quenching coefficient (NPQ (=Fm/Fm′-1)) and the photochemical quenching (qP) of hypoxic plants. This result further suggests that exogenous calcium could alleviate inhibition of the photochemical efficiency of cucumber by regulating the capacity of the heat-dissipation pathway, thereby reducing the negative impacts of hypoxic stress on the photosynthetic capacity of cucumber plants [38].

To understand the effect of different environmental stresses on photosynthesis, the measurement and analysis of fast chlorophyll a fluorescence is a useful and efficient method for the assessment of many external or intrinsic adverse effects on PSII photochemistry [39, 40], although the OJIP test renains a controversial interpretation. The typical polyphasic transient is changed under hypoxic stress (Fig. 4) and the positive ΔK-bands, ΔJ-bands, and ΔI-bands appear after illumination (Fig. 5). Previous studies indicated that the ΔK-bands and ΔJ-bands are associated with uncoupling of the OEC and the accumulation of Q_a_^−^ (i.e. inhibition of the re-oxidation of Q_a_^−^), respectively [24]. The efficient of the OEC (F_v_/F_0_) is the most sensitive component of photosynthetic electron transport [39]. The appearance of positive ΔK-bands in the fluorescence transients of stressed plants might indicate the OEC was damaged and the energetic connectivity between photosynthetic units was altered under hypoxic conditions [41]. The finding that the positive ΔK-bands were less pronounced in exogenous calcium-treated leaves than in hypoxic stressed ones might indicate the OEC was less damaged in the former than in the latter. The positive ΔI-bands (Fig. 5a) under hypoxic stress may suggested that hypoxia destroyed the acceptor side of PSII more severely than the donor side of PSII; the inactivation of the acceptor side might indicate the damaged of electron transport, according to previous studies [42]. Based on the Grouping Concept and JIP-test [40], the hypoxia-induced positive ΔI-bands (Fig. 5b) meant that the PSII units were destroyed into less grouped, then less electron and energy were being transported inside or between the PSII units. As the grouped conformation of PSII is more stable than the ungrouped one, the decreased grouping caused by hypoxic stress suggested that the stability of PSII units in stressed cucumber leaves had been lost and the PSII units became more fragile. As showed in Fig. 5, our study supported that the heterogeneity of the OJIP test was increased under hypoxic treatment.

In this study, the decrease in the fraction of active RCs (estimated as an increase of ABS/RC) was observed in hypoxic stressed plants (Fig. 6). The inactivated fraction of RCs or the increased apparent antenna size will lead to a decrease of this parameter. Accumulation of inactive RCs is related to the increased efficiency of absorbed light dissipation as heat (DI_0_/RC) (Fig. 6), indicating a higher level of the non-photochemical de-excitation process. To protect stressed leaves from photo-oxidative damage, plants disspute the excess absorbed light energy as heat. As indicated by decreases in the total performance index (PI_(abs)_), hypoxic stressed leaves had decreased Sm and DF_(ABS)_, increased TR_0_/RC and ABS/RC, and impaired photochemical and non-photochemical redox reactions (Fig. 6). Exogenous calcium reduced the values of TR_0_/RC, ABS/RC, and DI_0_/RC to control levels, indicating that exogenous calcium enhanced electron transport capacity of stressed leaves, thereby relieving the hypoxia-mediated damage of cucumber leaves.

## Conclusions

Hypoxic stress might impair the photosynthetic electron-transport chain from the donor side of PSII up to the reduction of end acceptors of PSI, thus limiting the production of reduction equivalents and the rate of CO_2_ assimilation. Exogenous calcium enhanced electron transport capacity and reduced hypoxic damage of cucumber leaves. We still need a further research to investigate what calcium dose mechanistically to cause all of this.

## Abbreviations

ABS/RC: Absorption flux per RC
*C*_i_: Intercellular CO_2_ concentration
DI_o_/RC: Dissipated energy flux per RC at *t* = 0
DO: Dissolved oxygen
ET_o_/RC: Electron transport flux per RC at t = 0
F_0_: Minimum fluorescence, when all PSII RCs are open
F_300μs_: Fluorescence intensity at 300 μs
F_I_: Fluorescence intensity at I-step (30 ms)
F_J_: Fluorescence intensity at J-step (2 ms)
F_m_: Maximum fluorescence, when all PSII RCs are closed
Fv/Fm: Maximum quantum yield of PSII
*g*_s_: Stomatal conductance
NPQ (=Fm/Fm′-1): Non-photochemical quenching coefficient
OEC: Oxygen-evolving complex
PI: Performance index
PI_(abs)_: Performance index (PI) on absorption basis
*P*_n_: Net photosynthetic rate
PPFD: Photon flux density
PSI: Photosystem I
PSII: Photosystem II
Q_a_: Primary quinone acceptor
Q_B_: secondary acceptor plastoquinone
qP: Photochemical quenching coefficient
RC: PSII reaction centre
RH: Relative humidity
ROS: Reactive oxygen species
Sm: Normalized total complementary are above the OJIP
*T*_r_: Transpiration rate
TR_o_/RC: Trapped energy flux per RC at *t* = 0
V_t_: Relative variable fluorescence at time t
WUE: Water use efficiency
φP_o_: Maximum quantum yield of primary photochemistry at t = 0
ΦPSII: Actual photochemical efficiency of PSII
